# The simultaneous recognition of multiple words: A process analysis

**DOI:** 10.3758/s13421-020-01082-w

**Published:** 2021-04-08

**Authors:** Anne Voormann, Mikhail S. Spektor, Karl Christoph Klauer

**Affiliations:** 1 Department of Psychology, University of Freiburg, Engelbergerstraße 41, Freiburg, 79106 Germany; 2 Department of Economics and Business, Universitat Pompeu Fabra, Barcelona, Spain; 3 Barcelona Graduate School of Economics, Barcelona, Spain

**Keywords:** Recognition memory, Continuous models, Discrete-state models, Cognitive modeling

## Abstract

In everyday life, recognition decisions often have to be made for multiple objects simultaneously. In contrast, research on recognition memory has predominantly relied on single-item recognition paradigms. We present a first systematic investigation into the cognitive processes that differ between single-word and paired-word tests of recognition memory. In a single-word test, participants categorize previously presented words and new words as having been studied before (old) or not (new). In a paired-word test, however, the test words are randomly paired, and participants provide joint old–new categorizations of both words for each pair. Across two experiments (*N* = 170), we found better memory performance for words tested singly rather than in pairs and, more importantly, dependencies between the two single-word decisions implied by the paired-word test. We extended two popular model classes of single-item recognition to paired-word recognition, a discrete-state model and a continuous model. Both models attribute performance differences between single-word and paired-word recognition to differences in memory-evidence strength. Discrete-state models account for the dependencies in paired-word decisions in terms of dependencies in guessing. In contrast, continuous models map the dependencies on mnemonic (Experiment 1 & 2) as well as on decisional processes (Experiment 2). However, in both experiments, model comparison favored the discrete-state model, indicating that memory decisions for word pairs seem to be mediated by discrete states. Our work suggests that individuals tackle multiple-item recognition fundamentally differently from single-item recognition, and it provides both a behavioral and model-based paradigm for studying multiple-item recognition.

**Supplementary Information:**

The online version contains supplementary material available at 10.3758/s13421-020-01082-w.

Recognition plays an important role in everyday life: We have to recognize faces of people, landmarks for navigation, or our bike in a group of bikes. Scholarly investigations of recognition memory typically use single-word recognition tasks in which participants first study word lists in a study phase and afterwards categorize presented words as previously studied (*old*) or not (*new*) in a recognition phase (e.g., Allen & Garton, 1968; Snodgrass & Corwin, 1988; Bröder & Schütz, 2009). In contrast, the everyday-life recognition context is typically considerably more complex. For example, we often first meet people separately, and later encounter them as members of a larger group, at which point we have to decide whom in the group we actually know and whom not, which people are ‘old’ and which are ‘new’. The question we seek to study here is how recognition decisions for multiple objects differ from the recognition of single objects. For this purpose, we used a paired-word recognition paradigm in which participants have to categorize two randomly paired words simultaneously.

Evidence from related domains suggest that these two kinds of decisions differ in important ways. Consider the case of witness questioning, in which sequential and simultaneous lineups have been compared. In a sequential lineup, each lineup member is presented separately and judgments are made after each presentation, similarly to single-word recognition. A simultaneous lineup presents all lineup members at the same time, which is more akin to multiple-word recognition. Interestingly, the identification rate is higher (and consequently, fewer targets are missed) in simultaneous than in sequential lineups while more lures are correctly rejected in sequential lineups (e.g., Steblay et al., 2001; Steblay et al., 2011). The difference between simultaneous and sequential lineups is often explained by the use of different strategies: Simultaneous, but not sequential, lineups support comparative judgments (Lindsay & Wells, 1985). Wixted and Mickes (2014) specified these differences further: Their diagnostic-feature detection hypothesis posits that in simultaneous lineups, only diagnostic features (i.e., features that are not shared by all lineup members) are used for discrimination, whereas in sequential lineups witnesses use all available features (diagnostic and non-diagnostic). Because non-diagnostic features are non-diagnostic of guilt, focusing on diagnostic features enhances the ability to discriminate innocent from guilty suspects.

Note that this account capitalizes on the fact that the different recognition decisions made or implied for each lineup member are by design not independent of each other.^1^ The knowledge that at most one perpetrator exists can validly inform other decisions, and in Wixted and Mickes’ (2014) account, it even informs participants’ focus on certain features at the expense of others. Here, we address the question whether multiple decisions to simultaneously presented objects, each of which can in principle be old or new, are independent.

If two independent stimuli are presented simultaneously, independent judgments could be expected, given an optimally tuned memory system and decision strategy. Considering human decision makers, one plausible idea would indeed be that people evaluate the two objects sequentially and independently, one object after the other. On the other hand, even in single-word recognition tasks, in which participants judge objects sequentially, there exists evidence that two subsequent judgments are not always independent from one another (e.g., sequential effects exist for certain trial combinations; Ratcliff & Starns, 2009). However, models describing single-word recognition typically do not include such dependencies. For example, signal detection theory, an account in which responses are based on the comparison of a continuous familiarity signal produced from the presented stimulus and an internal response criterion, assumes that each presented stimulus is evaluated independently (Wickens, 2002). Neither familiarity signals nor criterion values are affected by preceding recognition decisions. Similarly, threshold models, which model recognition decisions as based on discrete “detect” and “uncertainty” states and guessing processes, do not permit dependencies between stimuli (Snodgrass & Corwin, 1988). Neither the probabilities of entering one of the modeled mental states nor the guessing distributions are affected by preceding recognition decisions.

As the first empirical investigation of its kind, Greene and Klein (2004) implemented a paired-word recognition task and found that recognition performance to randomly paired objects *does* differ from recognition performance to single objects. More specifically, Greene and Klein compared the performance in paired-word trials of “pseudoparticipants”, calculated from participants’ responses in single-word trials, to the performance of real participants either deciding whether both words were old (‘both condition’) or whether at least one word was old (‘either condition’). They found performance differences for certain response combinations between pseudoparticipants and real participants, without differences in overall accuracy. Greene and Klein discussed, but did not decide between, a number of possible mechanisms that may have caused the differences, notably criterion shifts, sequential effects, use of associative information as well as a spill-over of evidence from one familiarity signal to the other.^2^

The goal of the present study is to provide a first systematic investigation into the cognitive processes that differ between the classical single-word recognition task and the paired-word recognition task. Additionally, we want to investigate whether dependencies within the recognition decisions for paired-word trials occur at all and if they do, how they can be characterized. Do they originate in dependencies between the signals retrieved from memory (mnemonic origin), in dependencies in the way in which these signals are used to arrive at old/new decisions (decisional origin), or in dependencies at both levels (mnemonic and decisional origin)? To address these issues, we extended the two previously mentioned models of recognition decisions, continuous and discrete-state models, (Kellen & Klauer, 2014) to the paired-word task, and endowed them with the capability to model the above-mentioned theoretical possibilities.

## Modeling recognition decisions in the paired-word task

### Continuous models

Recognition models based on signal detection theory (Swets et al., 1961) are arguably the most prominent examples of continuous models. They assume that recognition decisions are based on a continuous familiarity signal. Each time the familiarity of a stimulus exceeds a certain criterion *c* the stimulus is categorized as ‘old’ (Wickens, 2002), otherwise the response ‘new’ is given. The difference in the mean familiarity of previously studied words (*targets*) and new words (*lures*), $d^{\prime }$, reflects the memory sensitivity.

**Fig. 1:**
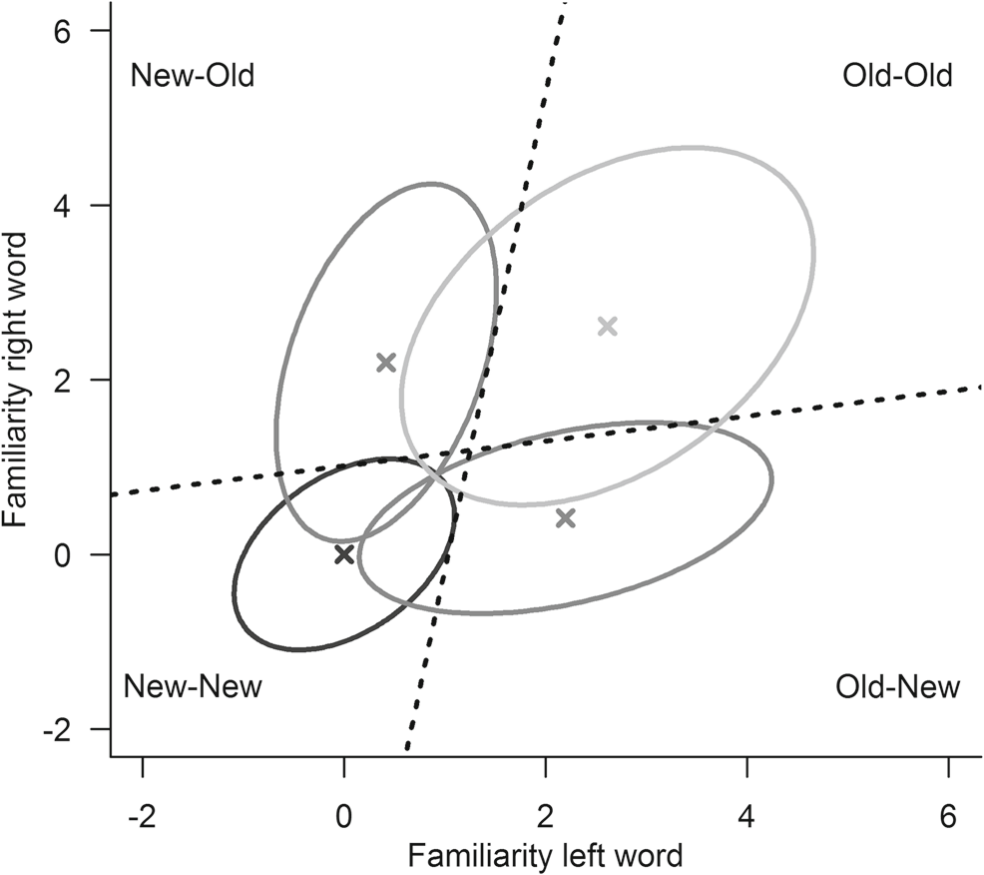
Illustration of the general recognition theory for paired-word recognition using the mean group-level posterior parameters of Experiment 2. Ellipses represent the different stimulus types: black for two new words, gray for one old word paired with one new word, and light gray for two old words. Dashed lines indicate the response criteria that separate the respective responses

In cases in which multiple words are presented simultaneously, a multidimensional version of signal detection theory, general recognition theory (GRT), can be applied (Ashby & Perrin, 1988; but see also Ashby & Soto, 2015). GRT was originally developed to characterize dependencies between different dimensions of perception, but can be adapted to test for dependencies within recognition decisions on both the mnemonic and decisional level (see Fig. 1; Ashby & Gott, 1988; Ashby & Townsend, 1986).

In a paired-word recognition task, two dimensions are considered (one for each word). To model dependencies at the mnemonic level, we permit spill-over between the two familiarity values elicited by the two members of a pair, leading (a) to covariation of the members’ familiarity signals across trials and (b) to overall shifts in the mean familiarity value of each member depending on whether the other member is an old or new word. Dependencies at the decisional level are incorporated by letting response criteria for each word depend upon the familiarity signal elicited by the other word of a given word pair.

### Discrete-state models

Discrete-state models assume that (possibly continuous) evidence from memory is discretized into a small number of mental states which then drive the recognition decision (Riefer & Batchelder, 1988). One prominent example is the two-high threshold model (2HTM; Snodgrass & Corwin, 1988). According to the 2HTM, a target (lure) enters a detect-old (detect-new) state with probability *d*_*o*_ (*d*_*n*_). With probability 1 − *d*_*o*_ (1 − *d*_*n*_), the test item enters a state of uncertainty, in which individuals can do little more than guess the answer ‘old’ with probability *g* and ‘new’ with probability 1 − *g* (Bröder & Schütz, 2009). Note that guessing can strategically use whatever contextual information is available to participants such as the base rates of old relative to new words in the test list or, in the present case, the mental state which the other pair member has entered.

**Fig. 2:**
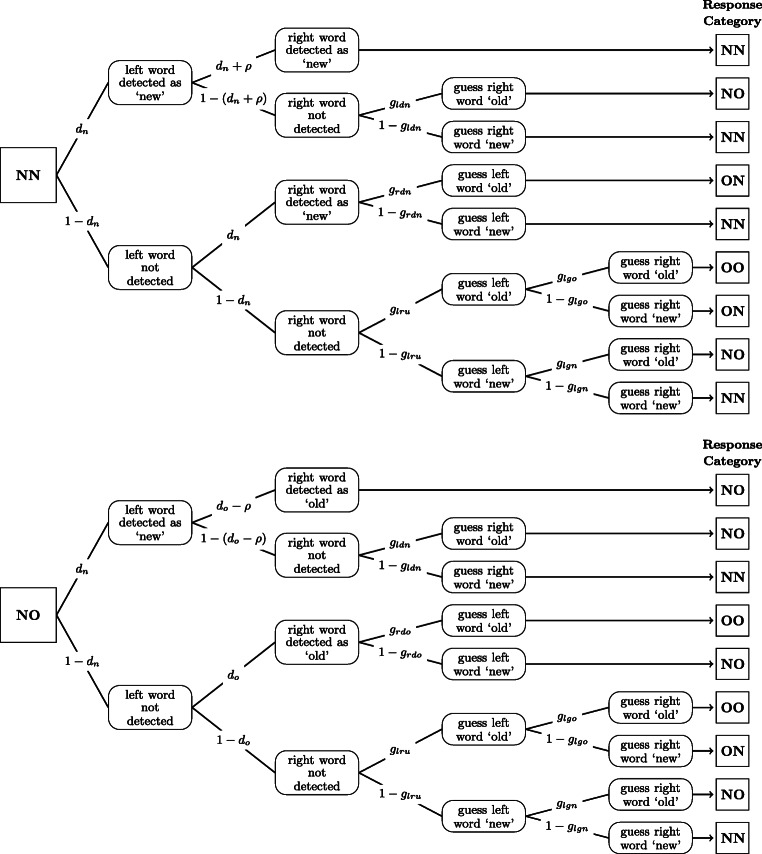
Extended two-high threshold model for paired-word recognition. Abbreviations for response categories and stimuli: NN—both words new; NO—left word new and right word old; ON—left word old and right word new; OO—both words old. The trees for pair types ON and OO are not shown. For a full description of the parameters, see Supplementary Material

We extended the 2HTM to multiple-word recognition tasks by assuming sequential decisions for the two presented words (see Fig. 2). To model dependencies in mnemonic processes, we permitted a spill-over of evidence between the two recognition decisions within the detection states. Additionally, the introduction of several guessing states dependent on the decision state of the other word covers dependencies of decisional origin.

Using extended versions of both a continuous and a discrete-state model does justice to an ongoing debate on whether recognition decisions are based on a continuous memory signal or mediated via discrete states (see, e.g., Pazzaglia et al., 2013; Province & Rouder, 2012; Kellen & Klauer, 2011; Batchelder & Alexander, 2013), with some arguing that the continuous versus discrete nature of information used is task-dependent (Kellen & Klauer, 2014; McAdoo et al., 2019).

## Experiment 1

The aim of the first preregistered study was threefold. First, we aimed to replicate Greene and Klein’s (2004) finding that there exists a difference between single- and paired-word recognition and to evaluate whether this is (partially) mediated through dependencies within paired-word recognition trials. The second aim was to assess the performance of the discrete-state and continuous model classes within this recognition paradigm, to achieve an optimal description of the mechanism involved. Third, we wanted to provide a model-based characterization of the behavioral differences at the level of cognitive processes. Such a characterization allows us to distinguish whether behavioral differences between single- and paired-word recognition reflect changes in decision strategies (i.e., how a memory signal is transformed into decisions, as modeled by the response criteria *c* or guessing parameters *g*), in the mnemonic process (i.e., better or worse memory of the items, as captured by differences in mean familiarity $d^{\prime }$ or detection probabilities *d*_*o*_), or in both. Additionally, it allows us to assess whether possible dependencies within paired-word trials reflect dependencies in mnemonic, decisional, or both processes.

To achieve these goals, we used a within-participant design in which individuals completed both single- and paired-word recognition trials to permit a comparison of the cognitive processes involved. We adapted the 2HTM and the GRT to be able to model both kinds of trials simultaneously. Within each model class, we rigorously selected the best-performing model to get an optimal description of the processes involved. Thereafter, we compared the respective winners with each other to evaluate which model is best suited for the joint description of single- and paired-word recognition.

### Method

Both studies were conducted in line with the ethical standards of the German Psychological Society and have been preregistered prior to data collection on the Open Science Framework (OSF). All preregistration materials, code to run the experiments in PsychoPy 2 (Peirce, 2007), raw data, data-analysis scripts, and model codes in Stan (Carpenter et al., 2017) can be found at https://osf.io/cdtep/. As the stopping rule for both experiments, we preregistered to terminate data collection once 80 valid data sets had been collected. Because we had no firm expectations regarding effect sizes, we decided to sample 40 participants per group for a total of 80 participants (not counting excluded participants), thereby doubling sample sizes from Greene and Klein (2004) who sampled *n* = 20 per group.

#### Participants

A total of 88 participants completed the experiment for partial course credit or a monetary reward. As per preregistration, we excluded eight participants because they were not native speakers of German or showed performance not significantly above chance level. The remaining 80 participants (15 male, 65 female) were mostly university students with different majors and ages between 18 and 50 years (*M* = 26.10, *SD* = 5.48).

#### Materials

The experiment’s word pool consisted of 644 German nouns taken from Lahl et al., (2009). Each study list comprised 68 items randomly drawn from the word pool. We used the first and last two items of each study list for initial warm-up trials for the test blocks that were later discarded. There were three different types of test blocks: single-word blocks, paired-word blocks, and mixed blocks.

Single-word blocks consisted of 64 single-word trials, 32 old words randomly drawn from the study list and 32 new words not previously studied. Four additional words, two old and two new, were used for warm-up trials. Paired-word blocks comprised 64 paired-word trials presenting two words left and right of the screen center. These trials could be composed of two old words (old–old), two new words (new–new), an old word on the left side and a new word on the right side (old–new), or vice versa (new–old). There were 16 trials of each type. The four initial warm-up trials consisted of one trial per pair type. Mixed blocks included 32 single-word trials (16 old and 16 new words) and 32 paired-word trials (eight trials per pair type). Here, warm-up trials consisted of an old and a new item as well as two paired-word trials of either the type old–old and new–new or old–new and new–old.

In single-word trials, words appeared in the center of the screen. In paired-word trials, a horizontal gap of 60 mm separated the two words.

#### Design and procedure

The study implemented two between-participants conditions to which the participants were randomly assigned. In the mixed condition (*n* = 40), participants encountered single-word and paired-word trials intermixed. In the pure condition (*n* = 40), participants alternated between pure blocks of single-word trials and pure blocks of paired-word trials with the kind of the first block (single-word or paired-word) counterbalanced. Both conditions consisted of four study–test cycles.

In each study phase, words were presented sequentially central on the screen. Each word appeared for 3 s and with 300 ms interstimulus interval between two words.

The trial sequence within test blocks was randomized, but started with the four designated warm-up trials. Prior to the presentation of a single-word stimulus, a fixation cross was presented centrally for 800 ms. Participants had to decide whether the stimulus was old or new. Prior to the presentation of a paired-word stimulus, two fixation crosses appeared on screen for 800 ms at the positions at which the words appeared. Participants had to decide whether the presented word pair was of the type old–old (OO), old–new (ON), new–old (NO), or new–new (NN).

Each stimulus was visible on the screen until a response was recorded. An inter-trial interval of 600 ms separated two test trials. Participants were instructed to respond as quickly and accurately as possible using the arrow keys. For single-word trials the upper arrow key served for indicating old words and the lower arrow key for indicating new words. For paired-word trials participants used the upper arrow key to indicate old-old pairs, the lower arrow key for new-new pairs, the left arrow key for old-new pairs and the right arrow key for new-old pairs. The appropriate response mapping was visualized on every trial in the lower right corner of the screen. Between two study-test cycles, participants were required to solve four simple arithmetic problems and were allowed to take self-paced breaks.

#### Model specification, selection, and comparison

To evaluate the differences between single- and paired-word recognition within GRT, we implemented different criteria and mean-familiarity values for words tested in single-word trials and for words tested in paired-word trials. It is typically assumed that there is more variance in the familiarity of old words than of new words due to attention fluctuation during the study phase (Kellen and Klauer, 2018). To account for this, we allowed the variance of the familiarity values of old words ($\sigma ^{2}_{\text {old}}$) to differ from the variance for new words, which could be set equal to one without loss of generality. As the study phase is equivalent for single-word and paired-word trials, *σ*_old_ was set equal for old words from both kinds of trials. GRT accommodates possible dependencies between the recognition processes for the members of a word pair in two ways: At the decisional level, criteria can be influenced by the familiarity value of the other pair member (via parameters *b*_*l*_ and *b*_*r*_); at the mnemonic level, the familiarity values of the two pair members can be correlated (parameter *ϱ*). The latter implies a covariation of mean familiarity values across pair types (NN, NO, ON, and OO) that was modeled by spill-over parameter *μ*_spill_ (for more detailed information, see Supplementary Material).

For the 2HTM, we implemented separate detection states for each word within the paired-word trials. While *d*_*o*_ was allowed to differ between single- and paired-word trials, it was restricted to be equal for the left and right word in paired-word trials. For identifiability reasons, we assumed *d*_*n*_ to be invariant between single- and paired-word trials. To account for possible dependencies within paired-word trials on the mnemonic level, we added a novel parameter *ρ* increasing (for *ρ* > 0) or decreasing (for *ρ* < 0) detection probabilities in OO and NN trials relative to ON and NO trials (see Fig. 2 and Supplementary Material). To account for dependencies on the decisional level, we permitted guessing to be influenced by whether the other word had entered a detect-old state, had entered a detect-new state, had been guessed old, or had been guessed new (for detailed information, see Supplementary Material).

Our analyses followed a two-step procedure. In the first step, we used nested model comparisons of restricted models *within* each class of models (GRT and 2HTM) to identify the locus of differences between single-word and paired-word recognition as well as the nature of dependencies in paired-word recognition. Specifically, following recommendations by Wagenmakers et al., (2010), we fitted all models with and without any combination of parameter restrictions of interest and computed Bayes factors between them. For the GRT, the restrictions of interest addressed the following questions (see Supplementary Material for more details on the different model parameters):

1. Can the sensitivities for old words be set equal across single-word and paired-word condition ($\mu _{\text {old}_{s}} = \mu _{\text {old}_{p}}$)?
2. Can the criteria in the single-word condition and for left pair members in the paired-word condition be set equal (*c*_*s*_ = *c*_*l*_)?
3. Is the mean spill-over *μ*_spill_ different from zero?
4. Is the correlation *ϱ* different from zero?
5. Is the decisional-dependence parameter *b*_*l*_ different from zero?
6. Is the decisional-dependence parameter *b*_*r*_ different from zero?

For the 2HTM, the restrictions address the following questions:

1. Can the detection probabilities for old words be set equal across single-word and paired-word condition ($d_{o_{s}}=d_{o_{p}}$)?
2. Can the guessing probabilities in the single-word condition and for left pair members given uncertainty about the right pair member be set equal (*g*_*s*_ = *g*_*lru*_)?
3. Is the mnemonic-dependence parameter *ρ* different from zero?
4. Do guessing probabilities for the left pair member differ depending upon whether the right pair member was detected to be old versus detected to be new (*g*_*rdo*_ = *g*_*rdn*_)?
5. Do guessing probabilities for the right pair member differ depending upon whether the left pair member was detected to be old versus detected to be new (*g*_*ldo*_ = *g*_*ldn*_)?
6. Do guessing probabilities for the right pair member differ depending upon whether the left pair member was guessed to be old versus guessed to be new (*g*_*lgo*_ = *g*_*lgn*_)?

In the second step, we pitted the best within-class models against each other to determine whether a continuous or a discrete-state model is best suited to characterize the behavior in the experiment.^3^

We implemented all models within an hierarchical Bayesian framework. For model selection, we relied on Bayes factors that allow us to quantify the evidence in favor of or against each model out of a set of models. In contrast to likelihood-ratio tests and information criteria such as the Akaike information criterion, Bayes factors control for the functional flexibility of a model in model comparisons rather than approximating model complexity using the number of free parameters as a proxy.

To compute Bayes factors for nested comparisons within each model class, we relied on the Savage–Dickey method, using effect coding for the to-be-tested differences between parameters (Wagenmakers et al., 2010). We specified priors on the effect parameters by using probability distributions for which 95*%* of the probability mass lies in an area corresponding to small to medium effects, as proposed by Wagenmakers et al. (2010, Appendix). For all other parameters, we used weakly informative priors (for a full description of the priors used, see the Supplementary Material). For within-class model comparison we fitted each nested model, retaining 60 000 samples from four chains after thinning with a factor of two. The first 10 000 samples were discarded as warm-up. We obtained Bayes factors for each nested model within each of the model classes relative to the most complex model, allowing us to calculate the posterior model probabilities. For each model in the set of candidate models, they reflect the posterior probability that this model rather than another of the candidate models has generated the data. They convey the same information as Bayes factors do in an easily interpreted format.

To compute Bayes factors comparing the best-performing models within each model class across classes, we estimated marginal likelihoods (using the bridgesampling package; Gronau et al., 2017) for the selected models, once including individual differences (i.e., with random effects for participants) for parameters constrained to be equal at the group level and once without such individual differences. If not already included, we added the respective full models to the comparison procedure. To achieve a relatively stable estimation of the marginal likelihood, a larger number of posterior samples is required (Gronau et al., 2017). Therefore, we based these analyses on 140 000 samples for each of the four chains with a thinning factor of four, discarding the first 40 000 samples as warm-up. Using marginal likelihoods to compare the selected models not only independently validates the procedure in the first step using another algorithm (Bayes factors as computed in the first step are ratios of marginal likelihoods). Additionally, it allowed us to select the single model that can best account for the behavioral data across model classes based on a Bayes factor comparison and to use this model to draw inferences about the underlying cognitive processes.

### Results

#### Data preparation and behavioral analyses

**Fig. 3:**
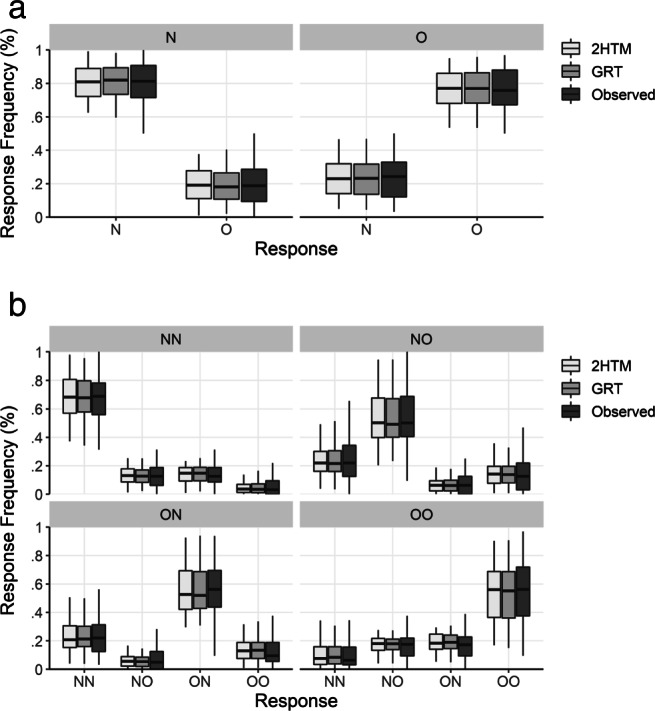
Median percentages and interquartile ranges of given responses (‘Observed’) to the respective pair types and the predicted frequencies of the two-high threshold model (2HTM) and general recognition theory (GRT) for single words (panel A) and paired words (panel B). N—word not studied (new); O—word studied (old); NN—both words new; NO—left word new, right word old; ON—left word old, right word new; OO—both words old. Outliers are not depicted

As per our preregistration, we excluded all trials with responses faster than 250 ms or slower than 10 s which led to an exclusion of 0.19 % of total trials. Figure 3 shows the response frequencies for each trial type for single- and paired-word trials.

**Fig. 4:**
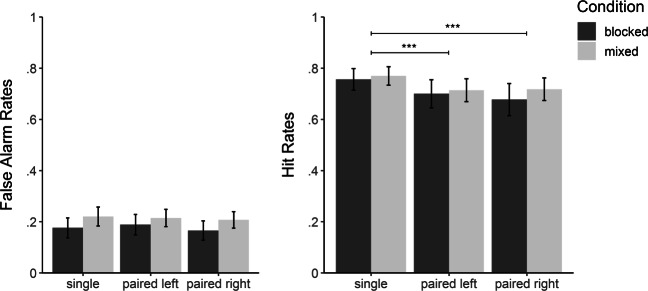
Hit and false-alarm rates for single-word trials (single) and paired-word trials split into words appearing on the left (paired left) and the right (paired right) side of the screen for each condition (blocked/mixed). Error bars reflect the respective standard errors. *** = *p* < .001

To assess whether there was a performance difference between single- and paired-word trials, we conducted two 2 × 3 mixed ANOVAs, one on hit rates and one on false-alarm rates with condition (pure vs. mixed) as between-participants factor, and trial type (single, paired-left, vs. paired-right) as within-participant factor (see Fig. 4 for mean values). Hit and false alarm rates were arcsine-transformed to correct for dependencies between the mean and standard deviation of rates. Where necessary, we used Greenhouse–Geisser corrected degrees of freedom. We neither found an effect of condition on hit rates, *F*(1,78) = 0.30, *p* = .588, ${\eta ^{2}_{p}} = .004$, 95*%* confidence interval of the effect (CI): [.00,.07], nor on false-alarm rates, *F*(1,78) = 3.12, *p* = .081, ${\eta ^{2}_{p}} = .04$, 95*%* CI: [.00,.16], suggesting that there are no substantial differences between mixed and pure blocks. More importantly, we found a significant difference between trial types within hit rates, *F*(1.85,144.2) = 22.04, *p* < .001, ${\eta ^{2}_{p}} = .22$, 95*%* CI: [.11,.34] but not within false-alarm rates, *F*(1.83,143.0) = 1.53, *p* = .222, ${\eta ^{2}_{p}} = .02$, 95*%* CI: [.00,.08]. Using paired-sample *t*-tests and a Bonferroni-corrected alpha level of .017, post-hoc analyses revealed that the effect within hit rates was driven by differences between single-words and paired-left words, *t*(79) = 5.32,*p* < .001, *d*_*z*_ = 0.36, 95*%* CI: [0.22,0.50], as well as paired-right words, *t*(79) = 5.47, *p* < .001, *d*_*z*_ = 0.38, 95*%* CI: [0.24,0.52], whereas there were no differences between paired-left and paired-right words, *t*(79) = 0.84, *p* = .401, *d*_*z*_ = 0.04, 95*%* CI: [− 0.06, 0.15]. In sum, the results reveal that recognition performance is impeded for paired words relative to single words.

In a second step, we tested for dependencies in responses within paired-word trials. We used the log-likelihood ratio statistic, *G*^2^, to assess whether the response probabilities of the sixteen paired-word response categories (e.g., respond ‘OO’ for a test pair of type ON) can be represented as the product of the probabilities of the implied responses for the left pair member and the right pair member. For example:

$$ \begin{array}{@{}rcl@{}} &&P(\text{respond `OO' for a test pair of type ON}) = \\ &&\quad P(\text{respond `O' for the left pair member given a left pair member of type O}) \times \\ &&\quad P(\text{respond `O' for the right pair member given a right pair member of type N}). \end{array} $$

We computed the *G*^2^ statistic for these independence restrictions relative to a saturated model for each participant and then summed the *G*^2^ statistics across participants. We evaluated its statistical significance using a bootstrapped *p*_*b*_ value based on 1 000 bootstrap samples to avoid biases with the asymptotic *χ*^2^ distribution of the test statistic due to many empty cells (see, e.g., Coolin et al., 2015). The results confirmed that the restriction of independent responses within paired-word trials cannot be upheld, $G^{2}_{\text {emp}}(640) = 857.10$, *p*_*b*_ < .001. This suggests that recognition decisions on paired words exhibit dependencies and mutually influence each other.

#### Model selection and comparison

Although behavioral analyses indicate dependencies within the responses to paired-words, they do not allow us to relate these behavioral dependencies to specific cognitive processes. To characterize the cognitive processes underlying paired-word recognition, we relied on model-based analyses. First, we selected the best-fitting models within each model class based on the posterior model probability and marginal likelihoods (see Table 1 for the results of the two best fitting models and the full model per model class).

**Table 1:** Experiment 1: Mean [Minimum; Maximum] logarithms of marginal likelihoods (LML) and posterior model probabilities (PMP) for the full models and the two best-fitting models for both model classes, general recognition theory (GRT) and the two-high threshold model (2HTM), once with and once without individual differences (noID) on the restricted parameters

GRT			2HTM		
Model	LML	PMP	Model	LML	PMP
*M*_GRT, Full_	-2644 [-2646; -2640]	.005	*M*_2HTM, Full_	-2636 [-2630; -2621]	.007
*M*_GRT, 1_	-2638 [-2640; -2633]	.238	*M*_2HTM, 1_	-2621 [-2622; -2618]	.237
*M*_GRT, 1−noID_	-2607 [-2608; -2604]		*M*_2HTM, 1−noID_	-2602 [-2604; -2599]	
*M*_GRT, 2_	-2640 [-2642; -2637]	.195	*M*_2HTM, 2_	-2622 [-2624; -2619]	.183
***M***_**GRT, 2**−**noID**_	**-2601** [**-2603; -2599**]		***M***_**2HTM, 2**−**noID**_	**-2590** [**-2593; -2588**]	

For GRT, the models have the following restrictions: *M*_GRT, Full_ represents the GRT model with all implemented parameters, *M*_GRT, 1_ describes a GRT model with the restrictions *b*_*l*_ = 0, *b*_*r*_ = 0, *c*_*s*_ = *c*_*l*_, and *ϱ* = 0 and *M*_GRT, 2_ includes the same restrictions as *M*_GRT, 1_ without the restriction of *ϱ* = 0. For 2HTM, the models include the following restrictions: *M*_2HTM, Full_ represents the 2HTM with all implemented parameters, *M*_2HTM, 1_ describes a 2HTM with the restrictions *g*_*s*_ = *g*_lru_, and *g*_lgn_ = *g*_lgo_, and *M*_2HTM, 2_ includes the same restrictions as *M*_2HTM, 1_ with the additional restriction of *ρ* = 0. Selected models are highlighted in bold

Within the GRT class of models, the best model attributed the difference between single-word and paired-word recognition to familiarity differences ($\mu _{\text {old}_{s}} - \mu _{\text {old}_{p}}~:~$ *M* = 0.42, *SD* = 0.11, 95*%* posterior interval [PI]: [0.24,0.66]), such that old single words elicit higher familiarity values ($\mu _{\text {old}_{s}}: M = 2.20$, *SD* = 0.27, 95*%* PI: [1.75,2.80]) than old paired words ($\mu _{\text {old}_{p}}: M = 1.78$, *SD* = 0.20, 95*%* PI: [1.44,2.24]). The dependencies within paired-word recognition are best explained by mnemonic dependencies: A spill-over of familiarity between the two words (*μ*_spill_ : *M* = 0.08, *SD* = 0.03, 95*%* PI: [0.02,0.15]) and a positive correlation between the two familiarity values of paired words (*ϱ* : *M* = .05, *SD* = .03, 95*%* PI: [−.01,.11]; the Supplementary Material shows the results for all parameters).

The best fitting model within the class of 2HTM attributed the difference between single-word and paired-word recognition to differences in the detection probabilities for old words ($d_{o_{s}}-d_{o_{p}}: M = .10 $, *SD* = 0.02, 95*%* PI: [.05,.15]), with a higher probability to detect old words in single-word trials ($d_{o_{s}}: M = .61 $, *SD* = 0.03, 95*%* PI: [.55,.68]) than in paired-word trials ($d_{o_{p}}: M = .52$, *SD* = 0.05, 95*%* PI: [.44,.59]). The dependencies within paired-word trials are due to guessing processes. More specifically, the probability to guess ‘old’ for the right word was smaller if the left word was new and correctly detected as ‘new’ (*g*_ldn_ : *M* = .30, *SD* = 0.05, 95*%* PI: [.20,.40]) than if the left word was old and correctly detected as ‘old’ (*g*_ldo_ : *M* = .41, *SD* = 0.05, 95*%* PI: [.32,.51]) with a mean difference of *M* = .11 (*SD* = 0.05, 95*%* PI: [.01,.20]). Likewise, the probability to guess ‘old’ was smaller for the left word if the right word was correctly detected as ‘new’ (*g*_rdn_ : *M* = .35, *SD* = 0.04, 95*%* PI: [.26,.43]) than if it was correctly detected as ‘old’ (*g*_rdo_ : *M* = .45, *SD* = 0.05, 95*%* PI: [.36,.56]) with a mean difference of *M* = .10 (*SD* = 0.05, 95*%* PI: [.01,.20]; for the results of all parameters, see Supplementary Material).

In a second step, we compared the models across the two model classes. The best discrete-state model provided a better account of the data than the best continuous model, BF_2HTM, GRT_ = 2.9 × 10^4^, meaning that the discrete-state model is 2.9 × 10^4^ times more likely than the continuous model conditional on the observed data.

These results suggest that recognition decisions within a paired-word recognition task are mediated through discrete states rather than based on a continuous memory strength signal. However, it is possible that neither of the two model classes are able to quantitatively reproduce the data, such that the 2HTM might only be the lesser of two evils. To rule out this possibility, we checked the absolute fit of the selected models to the data. Figure 3 juxtaposes the observed relative frequencies for each response and trial type and the predicted relative frequencies. The predicted frequencies are sampled from the posterior predictive distribution of frequencies for each selected model. As can be seen in the figure, the major response patterns are well accounted for by both the GRT and the 2HTM model. In addition, we computed posterior predictive checks of category frequencies and the variance–covariance structure of response categories using the *T*_1_ and *T*_2_ statistics, respectively, as described in Klauer (2010). These measures compare how often predicted data generated from the estimated model deviate more strongly than the observed data from the values expected under the estimated model. This comparison leads to a posterior predictive *p*-value (*ppp*), where an optimal match is reflected in *ppp* = .5. *T*_1_ addresses deviations of the model from the mean category frequencies for the 20 overall response categories and *T*_2_ addresses deviations from the variance–covariance matrix (variances and covariances computed across participants) of the 20 response category frequencies. This analysis supported our previous conclusions as the 2HTM provided an adequate, although somewhat improvable, absolute fit, $T_{1}^{\text {pred}} = 13.0$, $T_{1}^{\text {obs}} = 21.1$, *ppp* = .112, and $T_{2}^{\text {pred}} = 27.3$, $T_{2}^{\text {obs}} = 36.7$, *ppp* = .174, still somewhat better than the GRT: $T_{1}^{\text {pred}} = 13.0$, $T_{1}^{\text {obs}} = 24.3$, *ppp* = .045, $T_{2}^{\text {pred}} = 27.3$, $T_{2}^{\text {obs}} = 40.1$, *ppp* = .110.

The Bayes factor used for model comparison weighs model fit and model complexity to account for the fact that a good model fit is the less impressive the more flexible the model is. Given the comparable absolute fit shown in the posterior predictive *p*-values, the very high Bayes factor in favor of the 2HTM model means that the 2HTM provides a substantially more parsimonious account of the data than the GRT model (for assessments of the relative complexity of discrete-state models and continuous models of recognition memory, see also Klauer & Kellen, 2011, 2015.)

### Discussion

Experiment 1 aimed to assess how paired-word recognition relates to single-word recognition and which cognitive model provides the best account of both tasks. Behaviorally, we found that recognition in paired-word trials is worse than what would be expected from two separate single-word recognition decisions. Both cognitive models, the GRT and the 2HTM, attribute this difference to depressed mnemonic processes, suggesting that memory retrieval is impaired in paired-word trials.

A second behavioral finding is that there exist dependencies within paired-word trials. The two considered model classes attribute the dependencies to different sources. GRT accounts for them on the mnemonic level through spill-over effects and a correlation between the elicited familiarity signals. In contrast, the 2HTM attributes the dependencies to the decisional level in terms of an increased bias to guess ‘old’ for pairs in which the other word was correctly identified as old relative to pairs in which the other word was correctly identified as new.

While both models accounted well for the major patterns in the data, rigorous model comparisons strongly favored the best 2HTM model over the best GRT model. This means that the 2HTM model accounted for the data far more parsimoniously than the GRT model.

## Experiment 2

Experiment 1 focused on differences between single- and paired-word recognition and on an exploratory analysis of dependencies within paired-word trials. Experiment 2 used only paired-word trials in the recognition phase to eliminate potential influences of the presence of single-word trials. We aimed to validate the results of Experiment 1 on two levels: Behaviorally, we expected to replicate the general finding of the presence of dependencies within recognition decisions involved in paired-word trials. From a model-based perspective, we aimed to corroborate the results that discrete-state models capture the process of paired-word recognition better than continuous models. Additionally, we wanted to validate the qualitative structure of these dependencies, namely whether they occur on the mnemonic level (as continuous models suggested) or on the decisional level (as discrete-state models suggested).

### Method

A total of 82 participants that had not participated in Experiment 1 completed Experiment 2. As per preregistration (https://osf.io/k4m8f/), we excluded two participants due to recognition performance not significantly above chance levels. The remaining 80 participants (23 male, 57 female) were mostly students from Freiburg University with different majors and between 18 and 42 years old (*M* = 24.00, *SD* = 4.04). All participants received either a monetary reward or partial course credit for participation.

As in Experiment 1, participants went through four study-test cycles. Each study phase consisted of 68 trials, including two trials each as recency and primacy buffers. Within the recognition phase, participants performed 68 recognition decisions per cycle, including four initial warm-up trials. Departing from Experiment 1, all recognition blocks were pure paired-word blocks. In all other respects, we used the same materials and procedure as in Experiment 1.

We used the same models specified in our first experiment, omitting all parameters related only to single-word recognition. The analysis procedure was otherwise identical to Experiment 1.

### Results

#### Data preparation and behavioral analysis

**Fig. 5:**
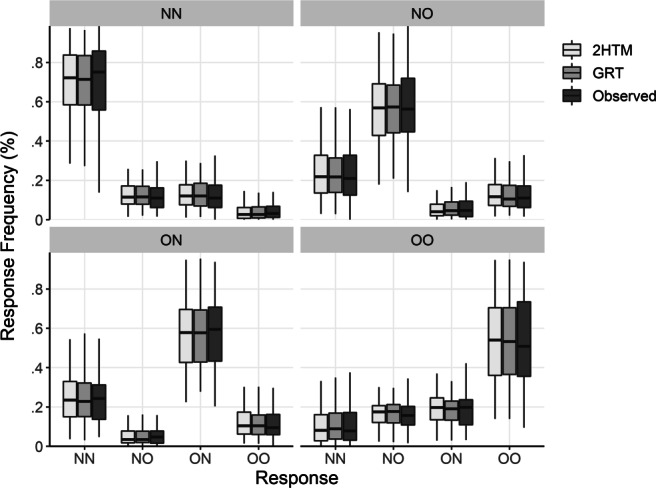
Median percentages and interquartile ranges of given responses (‘Observed’) to the respective four pair types and the predicted frequencies of the two-high threshold model (2HTM) and general recognition theory (GRT). NN—both words not studied (new); NO—left word new, right word studied (old); ON—left word old, right word new; OO—both words old. Outliers are not visualized

As in Experiment 1, we excluded trials with responses faster than 250 ms or slower than 10 s. This resulted into a total exclusion of 1.3 % trials. Figure 5 shows the response frequencies for each of the four pair types. Replicating the results of Experiment 1, a goodness-of-fit test revealed a significant violation of a model assuming independent reactions to both words, $G^{2}_{\text {emp}}(640) = 976.0, p_{b} < .001$ (based on 1,000 bootstrap samples).

#### Model selection and comparison

To provide a model-based characterization of the cognitive processes underlying the dependencies in paired-word recognition, we relied on model-based analyses. First, we selected the best-fitting models within each model class. Additionally, to the two best fitting models of each model class we included the full models of each model class for comparison (see Table 2 for the results of the model selection criteria). Within GRT, the full model was already one of the two best fitting models, so only two models are presented.

**Table 2:** Experiment 2: Mean [Minimum; Maximum] logarithms of marginal likelihoods (LML) and posterior model probabilities (PMP) for the full models and the two best-fitting models for both model classes, general recognition theory (GRT) and the two-high threshold model (2HTM), once with and once without individual differences (noID) on the restricted parameters

GRT			2HTM		
Model	LML	PMP	Model	LML	PMP
***M***_**GRT, Full**_	**-2531** [**-2534; -2523**]	**.652**	*M*_2HTM, Full_	-2527 [-2527; -2526]	.022
*M*_GRT, 1_	-2530 [-2533; -2525]	.235	*M*_2HTM, 1_	-2524 [-2524; -2523]	.522
*M*_GRT, 1−noID_	-2538 [-2539; -2538]		*M*_2HTM, 1−noID_	-2549 [-2550; -2548]	
			*M*_2HTM, 2_	-2525 [-2526; -2525]	.117
			***M***_**2HTM, 2**−**noID**_	**-2505** [**-2505; -2504**]	

The following restrictions are incorporated in the models: *M*_GRT, Full_ represents the GRT model with all implemented parameters, *M*_GRT, 1_ describes a GRT model with the restrictions *b*_*r*_ = 0, *μ*_spill_ = 0, and *ϱ* = 0, *M*_2HTM, Full_ represents the 2HTM with all implemented parameters, *M*_2HTM, 1_ describes a 2HTM with the restrictions *g*_ldn_ = *g*_ldo_, *g*_lgn_ = *g*_lgo_, and *ρ* = 0, and *M*_2HTM, 2_ includes the same restrictions as *M*_2HTM, 1_ excluding the restriction of *g*_lgn_ = *g*_lgo_. Selected models are highlighted with bold font

The full GRT model had the highest posterior model probability and therefore was the most likely model within the class of GRT. This model locates dependencies on both the mnemonic level, through spill-over effects (*μ*_spill_ : *M* = 0.29, *SD* = 0.07, 95*%* PI: [0.16,0.42]) and a correlation between the familiarity signals for the two members of each word pair (*ϱ* : *M* = .33, *SD* = .07, 95*%* PI: [.19,.46]), and on the decisional level, meaning that response criteria for each word are informed by the familiarity values elicited by the paired word (*b*_*l*_ : *M* = .11, *SD* = 0.03, 95*%* PI: [.04,.18]; *b*_*r*_ : *M* = .14, *SD* = 0.04, 95*%* PI: [.07,.21]). This model is, however, closely followed by the second-best fitting GRT model, which locates dependencies solely at the decisional level. Figure 1 represents the full GRT model constructed with mean group-level posterior parameter estimates.

Consistent with the results of Experiment 1, the best model within the class of discrete-state models attributes dependencies between paired words to the decisional level in terms of dependencies in guessing. More specifically, there was a lower tendency to guess ‘old’ when the right word was detected to be ‘new’ (*g*_rdn_: *M* = .38, *SD* = .04, 95*%* PI: [.29,.46]) rather than ‘old’ (*g*_rdo_: *M* = .51, *SD* = .05, 95*%* PI: [.41,.62]) with a difference of *M* = .13 (*SD* = .03, 95*%* PI: [.06,.20]). Likewise there was a lower tendency to guess ‘old’ when the left word was guessed to be ‘new’ (*g*_lgn_: *M* = .43, *SD* = .04, 95*%* PI: [.35,.51]) rather than ‘old’ (*g*_lgo_: *M* = .45, *SD* = .04, 95*%* PI: [.37,.53]) with a difference of *M* = .02 (*SD* = .04, 95*%* PI: [−.06,.10]).

In a second step, we compared the best within-class models with each other. The best 2HTM provided a better account of the data than the best model from the GRT class, BF_2HTM, GRT_ = 2.6 × 10^11^. This means that the best discrete-state model is 2.6 × 10^11^ times more likely given the data than the best continuous model.

Regarding absolute model fit, Fig. 5 juxtaposes the observed relative frequencies for each response and trial type and the predicted relative frequencies. As can be seen in the figure, both models capture the major responses patterns quite well. Considering the posterior predictive model checks in terms of the statistics *T*_1_ and *T*_2_, the full GRT model can account for the variance–covariance structure adequately, $T_{2}^{\text {pred}} = 31.7$, $T_{2}^{\text {obs}} = 37.8$, *ppp* = .319, whereas the ability to reproduce mean category frequencies falls off, $T_{1}^{\text {pred}} = 11.1$, $T_{1}^{\text {obs}} = 18.9$, *ppp* = .110. The 2HTM shows the same pattern as the GRT: It recovers the variance–covariance structure quite well, $T_{2}^{\text {pred}} = 31.9$, $T_{2}^{\text {obs}} = 29.3$, *ppp* = .588, whereas the recovery of the category frequencies falls off, $T_{1}^{\text {pred}} = 11.1$, $T_{1}^{\text {obs}} = 25.7$, *ppp* = .018. Again, given the comparable absolute fit, the very high Bayes factor in favor of the 2HTM model means that the 2HTM achieves this fit substantially more parsimoniously than the GRT model.

### Discussion

In Experiment 2 we aimed to validate the main results of our first experiment. Firstly, we replicated the main behavioral finding of Experiment 1 for paired-word trials, namely a dependency in recognition decisions on pairs of words. Secondly, we aimed to replicate the model-based results. Again, quantitatively, we found the discrete-state model to account better for the data than the continuous model. Thus, recognition decisions on paired words seem to be mediated through discrete states. Additionally, the discrete-state model again explained the dependencies within joint judgments of paired-words in terms of dependencies in guessing processes. The results within the continuous model are less consistent. In the second experiment, it located dependencies both on the mnemonic level, as in Experiment 1, and, unlike in Experiment 1, also on the decisional level.

## General discussion

The present study aimed to provide a first systematic investigation of differences between single-word and paired-word recognition tasks. We found two main behavioral results: Performance in paired-word recognition was lower than what would be expected from two separate single-word recognition decisions and, more importantly, recognition decisions in paired-word settings were not independent from another. To obtain a mechanistic understanding of the cognitive processes involved in these behavioral effects, we relied on computational modeling in which we compared the two dominant classes of recognition-memory models, continuous and discrete-state models, with each other. We found that a discrete-state model accounted for the data better than a continuous model. The winning model attributed differences between single- and paired-word recognition to differences in detection probabilities and dependencies between paired-word recognition decisions to guessing processes that depend on the decision state of the other stimulus.

### Mechanisms differing between single- and paired-word recognition

Consider first the differences in recognition performance between single-word and paired-word recognition tests. Both the 2HTM and the GRT attributed these to the mnemonic level in terms of higher detection probabilities and higher sensitivities, respectively, for the single-word test. Given that the study phase was the same for both kinds of test, this implies that retrieval attempts were more efficient for single-words than for paired-words.

Existing global matching models of memory (e.g., MINERVA2, SAM), although not adapted for multiple-item recognition, provide a framework for interpretation. Those models explain a successful recognition through a sufficient match between a memory probe and existing memory traces (see, e.g., Hintzman, 1988; Gillund and Shiffrin, 1984). In the paired-word trials, the probe is likely to encode features of, and associations between, both words with the potential to lower the overall match between the probe and memory traces of words studied in isolation. Thus, context effects on either the representational level or an associative level might account for the differences in detection probability and sensitivity. An adaptation of global-matching models for multiple items augmented by a model of the decision stage capturing dependencies could prove to be an appropriate model of multiple-item recognition and help to verify this assumption, but that is clearly a question for future investigations.

In addition to the mnemonic differences between single- and paired-word recognition, dependencies within recognition decisions to paired-words might have impaired performance on paired-word trials. Such dependencies occurred in both experiments. The discrete-state model accounts for these dependencies through a biased guessing process in the state of uncertainty whereas the assumption of independent detection processes for both words of a pair could be upheld. Although it can be useful to use all information available in a state of uncertainty, such a process is suboptimal in our setting, where the recognition decision to either word had no informational value or validity for the decision about the other word. However, it is interesting that those biases only occur in a state of uncertainty and do not influence detection. Thus, as long as there is enough evidence to reach a detection state for either word, the decision seems not to be influenced through irrelevant and potentially misleading information. Only in a state of uncertainty in which no information is available about the word to be judged (Kellen & Klauer, 2018), our memory system appears to be swayed by suboptimal guessing mechanisms.

The results for the continuous model paint a different and, across experiments, less consistent picture. GRT accounts for the observed dependencies on both the mnemonic level through a spill-over of evidence (Exps. 1 and 2) and the decisional level through an adaptive criterion adjustment based on the level of familiarity of the other presented item (Exp. 2). Thus, dependencies within the framework of GRT could be interpreted in terms of spill-over or cognitive leakage (as mnemonic mechanism), as Greene and Klein (2004) already surmised and/or sequential effects on criterion settings (as a decisional mechanism), which are, as already mentioned above, also known to occur within single-word recognition (see Ratcliff & Starns, 2009). Based on the results of the continuous model, it is also reasonable to assume an interaction between mnemonic and decisional mechanisms influencing paired-word recognition decisions. However, as discussed later, the quantitative comparison between this continuous account and the account delivered by discrete-state models strongly favored the latter over the former for the present data.

Discussing the dependencies within paired-word trials, the question might arise whether participants are able to discriminate the different sources of memory evidence at all or whether they instead rely on ensemble coding, the selection of a response based on the overall elicited memory signal (Dubé et al., 2019). A strong prediction of such a joint-signal strategy is that individuals would not be able to differentiate between trials in which an old word appeared on the right side and a new word on the left side or vice versa. Visual inspection of Figs. 3 and 5, combined with the results of previous studies using a comparable response scheme (see, e.g., Buchler et al., 2011; Buchler et al., 2008), reveals however that participants are well able to discriminate the different sources of evidence. Hence, a joint-memory-signal strategy cannot account for the observed dependencies in paired-word trials.

### Discrete and continuous accounts of paired-word recognition

Considering the comparison between discrete-state accounts and accounts in terms of continuous models, the best models from each model class accounted well for the major response patterns in the data as shown in Figs. 3 and 5. Nevertheless, model selection using Bayes factors revealed that discrete-state models strike the substantially better compromise between fit and parsimony. So far, the evidence suggests that in a simple paired-word recognition task responses are mediated through discrete states and are not based on a continuous memory signal.

Hence, in the case of joint recognition decisions to multiple objects, there seems to be no benefit of relying on continuous memory evidence. Nevertheless, this does not necessarily imply that all recognition decisions arise purely from continuous or discrete mechanisms. Rather, the processes which give rise to all-or-none states of detection may well rely on an underlying continuous signal of memory that is discretized as determined by task demands, response formats, and instructions (Kellen & Klauer, 2014; McAdoo et al., 2019; 2018).

### Comparison to Greene and Klein (2004)

The present experiments were in part motivated by Greene and Klein’s (2004) experiments and their finding that single-word performance does not predict paired-word performance. Like in the present experiments, Greene and Klein (2004) contrasted single-word recognition and paired-word recognition. Unlike in the present experiments, however, participants in their paired-word conditions were not asked to evaluate and indicate the old/new status of each pair member, but were given a number of more derived response instructions such as to judge whether both words were old or not (“both” condition) or whether at least one item was old or not (“either” condition). This renders a direct comparison of the present studies and Greene and Klein’s (2004) experiments difficult. Note, however, that the purpose of the present research was not to provide a close replication of Greene and Klein (2004), but to provide a process-oriented account of possible differences between single-word and paired-word recognition, keeping response instructions as similar as possible between the two conditions.

It is nevertheless instructive to compare our findings with Greene and Klein’s (2004) results in some more detail. Greene and Klein (2004) used an individual’s observed single-word responses to predict their responses to word pairs by assuming that responses to word pairs stem from two independent single-word recognition decisions.^4^ These predictions were labeled as stemming from “pseudoparticipants”. Major findings were (a) that more “both” responses and fewer “either” responses were made by real participants than by pseudoparticipants for each trial type, whereas (b) overall accuracy did not differ significantly between pseudoparticipants and real participants (see Table 3).

**Table 3:** Proportion and expected proportion (pseudoparticipants) of positive responses per pair type as well as overall accuracy for Greene and Klein’s (2004) Experiment 2 and Experiment 1 of this manuscript separately for “both” and “either” conditions

		Test pair type			
Condition	Participants	Old–Old	Mixed	New–New	*H* − *FA*
Greene & Klein (2004) Exp. 2					
“Both”	Real	.70	.53	.17	.29
	Pseudo	.53	.10	.03	.45
“Either”	Real	.80	.65	.18	.52
	Pseudo	.91	.79	.39	.44
Experiment 1					
“Both”	Real	.55	.13	.06	.44
	Pseudo	.60	.15	.05	.49
“Either”	Real	.89	.76	.31	.49
	Pseudo	.93	.81	.34	.51

Old–Old—pairs consisting of two old words; Mixed—pairs consisting of an old and a new word; New–New—pairs consisting of two new words; H − FA—difference between hit and false alarm rates

We constructed predictions for “both” and “either” conditions from the single-word data of our Experiment 1 exactly like in Greene and Klein (2004). These pseudoparticipant data should be comparable to Greene and Klein’s pseudoparticipant data given that both are based on traditional old/new response formats and instructions and computed in the same manner. For the data from real participants, we recoded participants’ responses. Thus, for the “both” condition, OO responses were coded as “both” response; for the “either” condition, ON, NO, and OO responses were coded as “either” response. Obviously, these data are less comparable with Greene and Klein’s (2004) data from real participants, given that we did not actually use “both” and “either” instructions and response formats.

Table 3 presents these data for our Experiment 1 and Greene and Klein’s (2004) Experiment 2.^5^ Three findings are noteworthy in this comparison:

1. Our pseudoparticipants and Greene and Klein’s (2004) show similar response frequencies, as might be expected.
2. Results for real participants diverge between Greene and Klein’s (2004) and our data.
3. The differences in overall accuracy between paired-word trials and single-word trials (i.e., between real and pseudoparticipants) are, if anything, smaller in our data than in Greene and Klein’s data (see column *H* − *FA*).

Finding 1 suggests that our data are consistent with Greene and Klein’s data when similar conditions are compared. Finding 2, in combination with Finding 1, suggests that the “both” and “either” response instructions introduce additional effects that are not induced by the old–new instructions used here. This is an interesting issue for further research. Considering Finding 3, note that simple indices such as *H* − *FA*, the difference between hits and false alarms, or $d^{\prime }$ do not succeed in disentangling discriminatory ability from response biases in the case of paired-word recognition and “both” and “either” response coding. Finding 3 exemplifies that as a consequence, differences in underlying discriminatory ability between single-word and paired-word trials as documented for our data may be masked. Disentangling the multiple response biases and old-new discriminations implied in paired-word recognition requires the more fine-grained response categories employed here in combination with appropriately extended models. Data collected with finer grain and models accommodating possible effects at the mnemonic level and the decisional level are furthermore the resources required to discriminate between different mechanisms accounting for the observed differences between single-word and paired-word recognition, where Greene and Klein (2004) could only speculate.

### Conclusion

Taken together, our results bear important implications for the study of recognition memory. An implicit assumption underlying most of recognition-memory research is that multiple-item recognition decisions can be treated as a sequence of independent single-item recognition decisions. Compare that situation with preferential decision-making research, in which axiomatic approaches explicitly formulate such independence assumptions (e.g., Luce, 1959) that can then be subject to rigorous investigations (see, e.g., Rieskamp et al., 2006, for an overview). The results of both our experiments strongly challenge such a notion in recognition memory as well, as supported by behavioral and model-based analyses.

Coming back to our introductory example, having met people separately, recognition of these people will be better if we meet them again separately rather than as members of a group. If we do meet them in the context of a group of people in a state of uncertainty, our recognition decisions about one person will inform the judgment of the other persons, irrespective of whether there exists a link or not. At least where recognition is involved, each time several independent decisions have to be made simultaneously, they can be expected to interact. Given the theoretical links to other domains, there is little reason to assume that decisional dependencies as documented here are confined to the domain of recognition memory: They may be found in simultaneous decisions under uncertainty, whatever is the subject matter of these decisions.

## Electronic supplementary material

Below is the link to the electronic supplementary material.

(PDF 288 KB)

## Data Availability

We preregistered all experiments prior to data collection on the Open Science Framework (OSF). All preregistration material, code to run the experiments, raw data, data-analysis scripts, and model codes can be found on the OSF (Experiment 1: https://osf.io/cdtep/; Experiment 2: https://osf.io/k4m8f/).

## Footnotes

1 Similar remarks pertain to other recognition paradigms in which multiple items are presented at test such as the two alternative forced-choice task or ranking tasks (Kellen & Klauer, 2018).

2 Note that all of the mechanisms used for explanation except for a spill-over of evidence have been argued to occur in the context of single-word recognition as well (e.g., Ratcliff & Starns, 2009; Marken & Sandusky, 1974; Rhodes & Jacoby, 2007; Homa, 1973).

3 We focused on the comparison of GRT and the adapted 2HTM because those models nest all models mentioned in the preregistration. For example, the “detection decrement model” mentioned in the preregistration is a version of GRT and 2HTM in which $d^{\prime }$ and *d*_*o*_, respectively, differ between single and paired words, while decision parameters stay constant for single and paired words and parameters capturing the dependencies within paired-words are set to zero.

4 For example, the predicted hit rate for “both” responses to old–old pairs was the square of the single-word hit rate, and the predicted hit rate for “either” responses to old–new pairs was the product of the single-word hit rate and the single-word correct-rejection rate.

5 The data from Greene and Klein’s (2004) Experiment 1 are similar; their Experiment 3 used yet other response instructions.
