# Supplementary material for: The simultaneous recognition of multiple words: A process analysis
Source: Mem Cognit. 2021 Apr 8;49(4):787–802. doi: 10.3758/s13421-020-01082-w (PMC8081710; doi:10.3758/s13421-020-01082-w)
Supplement: Supplementary file 1 — (PDF 288 KB) [file 13421_2020_1082_MOESM1_ESM.pdf]

Supplement: The simultaneous recognition of multiple objects: A process analysis

Anne Voormann[1], Mikhail S. Spektor[1, 2, 3], and Karl Christoph Klauer[1]

[1]University of Freiburg

[2]Universitat Pompeu Fabra

[3]Barcelona Graduate School of Economics

Supplementary Material

Model Specifications

Below we specify the models we implemented to evaluate the difference between single-word and paired-word recognition as well as the interaction of decisions within paired-word trials.

Continuous model

To model single-word recognition decisions based on a continuous memory signal, we used signal detection theory (SDT; Swets et al., 1961), corresponding to a one-dimensional variant of the general recognition theory (Ashby & Townsend, 1986). In SDT, it is assumed that the familiarity for targets and lures follows Gaussian distributions, with targets $\sim \mathcal{N}(\mu_{\text{old}_s}, \sigma_{\text{old}})$ and lures $\sim \mathcal{N}(\mu_{\text{new}}, \sigma_{\text{new}})$. Without loss of generality, identifiability can be established by fixing μ_{new} to 0 and σ_{new} to 1. The distance between the modes of the familiarity signal for targets and lures is represented by μ_{old_s}. Within SDT an 'old' response is given if the familiarity for a certain trial exceeds a criterion c_s, otherwise the answer 'new' is given. The probability of a hit H, the correct response to a target, is given by $Pr(\text{H}) = 1 - \phi(\frac{c_s - \mu_{\text{old}_s}}{\sigma_{\text{old}_s}})$, and the probability of a false alarm FA, the incorrect answer to a lure, is given by $Pr(\text{FA}) = 1 - \phi(c_s)$, where ϕ is the cumulative distribution function of a standard normal distribution.

To model paired-word recognition, we used GRT (see Ashby & Townsend, 1986). We considered two continuous familiarity dimensions, one for left and one for right words. The familiarities for each of the pair types (new–new, new–old, old–new and old–old) follow bivariate normal distributions with means $\boldsymbol{\mu}_i$ and a variance–covariance matrix Σ. The vector of means for the different pair types is as follows: $\boldsymbol{\mu}_{\text{new–new}} = (\mu_{\text{new}}, \mu_{\text{new}})$; $\boldsymbol{\mu}_{\text{new–old}} = (\mu_{\text{spill}}, \mu_{\text{old}_p})$; $\boldsymbol{\mu}_{\text{old–new}} = (\mu_{\text{old}_p}, \mu_{\text{spill}})$; $\boldsymbol{\mu}_{\text{old–old}} = (\mu_{\text{old}_p} + \mu_{\text{spill}}, \mu_{\text{old}_p} + \mu_{\text{spill}})$, where μ_{new} represents the mean familiarity for lures, μ_{old_p} the mean familiarity for targets, and μ_{spill} spill-over effects that are added to the respective mean. Those spill-over effects are defined as $\mu_{\text{spill}} = p \times \mu_j$, where μ_j represents the mean familiarity of the other dimension and p ranges between -1 and 1. Without loss of generality, μ_{new} is fixed to 0 as in SDT. Within each pair type, spill-over

occurring between the two individual elicited familiarity signals are captured by the correlation ϱ of the bivariate familiarity distributions. Their variance-covariance matrices can be determined for each pair type by considering the respective combination of σ_{new}, standard deviation for lures, σ_{old}, standard deviation for targets, and ϱ. As for the SDT and without loss of generality, σ_{new} is fixed to 1. To link the model for single- and paired-word recognition, we restricted σ_{old} to be equal for single- and paired-word trials.

Two different response criteria have to be implemented, one for each dimension. They take the shape of straight lines X_{c_l} and X_{c_r} in the bivariate familiarity space, partitioning that space into four response regions (see Figure 1 in the body of the text), corresponding to the four response options. To allow for dependencies between the items of a pair at the decisional level, we allowed both slopes and intercepts to vary independently: $X_{c_l} = b_l \times f_r + c_l$ and $X_{c_r} = b_r \times f_l + c_r$, where c represents the considered criterion if the other word is an average lure, f the amount of familiarity accumulated for the other dimension, and b the degree of decisional dependencies. We restricted b to the interval [-1, 1].

Discrete-state model

Within the class of discrete-state models, recognition decisions are made either in detection states or, if detection failed, in states of uncertainty. The two-high threshold model (2HTM) for single-word recognition specifies the probability of a hit H as $Pr(\text{H}) = d_{o_s} + (1 - d_{o_s}) \times g_s$, where d_{o_s} is the probability of a target being detected as 'old' and g_s is the probability of guessing 'old' if detection fails $(1 - d_{o_s})$. A hit either results from correctly detecting a target as being 'old' or from correctly guessing 'old'. The probability of a false alarm FA is given by $Pr(\text{FA}) = (1 - d_n) \times g_s$, where d_n is the probability of detecting a lure as 'new'.

We extended the 2HTM to paired-word trials by assuming two separate decisions. To allow for dependencies between the items of a pair at the decisional level, we allowed all uncertainty states to have different guessing probabilities. For example, the

probability of a new–old pair being correctly classified is given by

$$
\begin{aligned}
Pr(\text{`new–old'}|\text{new–old}) = {} & d_n \times (d_{o_p} - \rho) \\
& + d_n \times \Big(1 - (d_{o_p} - \rho)\Big) \times g_{ldn} \\
& + (1 - d_n) \times d_{o_p} \times (1 - g_{rdo}) \\
& + (1 - d_n) \times (1 - d_{o_p}) \times (1 - g_{lru}) \times g_{lgn}.
\end{aligned}
$$

A correct answer can be given through four different paths: 1) detecting the left word correctly as 'new' (d_n) and the right word as 'old' ($d_{o_p} - \rho$, parameter ρ is explained below), 2) detecting the left word as 'new' and guessing 'old' for the right word ($(1 - (d_{o_p} - \rho)) \times g_{ldn}$), 3) guessing 'new' for the left word ($(1 - d_n) \times (1 - g_{rdo})$) and detecting the right word as 'old', or 4) guessing 'new' for the left word ($(1 - d_n) \times (1 - g_{lru})$) and 'old' for the right word ($(1 - d_{o_p}) \times (g_{lgn})$). The full model has a total of 10 parameters in the paired-word part: d_{o_p} (d_n) probability of detecting a target (lure), g_{lru} probability of guessing 'old' for the left word given both words are in a state of uncertainty, g_{rdo} (g_{rdn}) probability of guessing 'old' for the left word given the right word was detected as 'old' ('new'), g_{ldo} (g_{ldn}) probability of guessing 'old' for the right word given the left word was detected as 'old' ('new'), g_{lgo} (g_{lgn}) probability of guessing 'old' for the right word given the left word was guessed to be 'old' ('new'). For comparability with the GRT, we added the parameter ρ to allow for dependencies within mnemonic processes: If the left word was detected, ρ is added to the detection probability of the right word if the pair is of type new–new or old–old, else it is subtracted. Behaviorally, this can be understood as a facilitation to detect the right word if the left word was already detected and both words belong to the same kind (target or lure). Because detection probabilities have to range in the interval from 0 to 1, the addition was actually done on a probit scale with probit-performed parameter values for the detection probabilities and parameter ρ. Parameter ρ can be positive, corresponding to positive dependencies between old and new detect states as just described, as well as negative, corresponding to negative dependencies.

To link single with paired-word recognition, we restricted d_n to be equal for single- and paired-words. As an additional benefit, this makes the estimation of both d_o and d_n possible within single-word recognition, which is usually not possible due to identifiability issues.

Priors

The models were specified hierarchically with separate parameters for overall means and for random effects by participants. A complete list of priors and individual-level parameter transformations is provided in Table S 1. Mean differences between corresponding parameters of single-word and paired-word recognition trials as well as parameters encoding dependencies between these trials were effect-coded with priors for the effect parameters specifying medium-sized effects a priori (Wagenmakers et al., 2010).

Parameter estimates of the best-fitting models from Experiments 1 and 2

Table S 2 reports the parameter estimates of the best-fitting models from Experiments 1 and 2.

Table S 1

Priors, transformations, and meanings for each parameter of both model classes (Class), general recognition theory (GRT) and two-high threshold model (2HTM)

Class	Parameter	Prior	Transformation	Meaning
GRT	Σ	Cauchy(0, 2.5)		SDs of person-level parameters
	Ω	LKJ(df=4)		Correlation matrix of person-level parameters
	δ	$\text{MVN}(\mathbf{0}, \Sigma^t\Omega\Sigma)$	mixed[a]	Person-level deviations from group-level means
	μ_{mean}	$\mathcal{N}_{T[0,\text{inf})}(0, 2)$		Mean familiarity for studied words
	μ_{diff}	$\mathcal{N}(0, 0.3)$	$exp(X)$	Effect of test type (single vs. paired) on mean familiarity of studied words (on a log scale)
	p	$\mathcal{N}(0, 0.2622003)$	$-1 + 2 \times \phi(X)$	Spill-over effect
	ϱ	$\mathcal{N}(0, 0.2622003)$	$-1 + 2 \times \phi(X)$	Correlation between the two familiarity dimensions
	b_l, b_r	$\mathcal{N}(0, 0.2622003)$	$-1 + 2 \times \phi(X)$	Adjustment of threshold based on other item's familiarity
	c_{mean}, c_r	$\mathcal{N}(0, 1)$		Mean threshold location for single and left paired words as well as right paired words
	c_{diff}	$\mathcal{N}(0, 0.5244005)$		Effect of test type (single vs. left paired words) on c_{mean}
	σ_{old}	$\mathcal{U}(0, 2)$	$1 + X$	SD of studied words' familiarity
2HTM	Σ	Cauchy(0, 2.5)		SDs of person-level parameters
	Ω	LKJ(df=4)		Correlation matrix of person-level distributions
	δ	$\text{MVN}(\mathbf{0}, \Sigma^t\Omega\Sigma)$	$\phi(X)$	Person-level deviations from group-level means
	d_o	$\mathcal{N}(0, 1)$	$\phi(X)$	Probability of detecting studied words
	$d_{o_{\text{sp}}}$	$\mathcal{N}(0, 0.5244005)$	$\phi(X)$	Effect of test type (single vs. paired) on the probability of detecting studied words
	d_n	$\mathcal{N}(0, 1)$	$\phi(X)$	Probability of detecting non-studied words
	$g_{\text{s, lru}}$	$\mathcal{N}(0, 1)$	$\phi(X)$	Probability of guessing 'old' for single words and left-paired words, conditional on being in a state of uncertainty
	$g_{\text{diff}_{\text{sp}}}$	$\mathcal{N}(0, 0.5244005)$	$\phi(X)$	Effect of test type (single vs. paired) on $g_{\text{s, lru}}$
	g_{ld}	$\mathcal{N}(0, 1)$	$\phi(X)$	Probability of guessing 'old' for the right word if the left word was detected
	$g_{\text{ld}_{\text{no}}}$	$\mathcal{N}(0, 0.5244005)$	$\phi(X)$	Difference in the probability of guessing 'old' for the right word if the left word was detected as 'new' vs. 'old'
	g_{rd}	$\mathcal{N}(0, 1)$	$\phi(X)$	Probability of guessing 'old' for the left word if the right word was detected
	$g_{\text{rd}_{\text{no}}}$	$\mathcal{N}(0, 0.5244005)$	$\phi(X)$	Difference in the probability of guessing 'old' for the left word if the right word was detected as 'new' vs. 'old'
	g_{lg}	$\mathcal{N}(0, 1)$	$\phi(X)$	Probability of guessing 'old' for the right word if the left word was not detected
	$g_{\text{lg}_{\text{no}}}$	$\mathcal{N}(0, 0.5244005)$	$\phi(X)$	Difference in the probability of guessing 'old' for the right word if the left word was guessed 'new' vs. 'old'
	ρ	$\mathcal{N}(0, 0.2622003)$	$\phi(X)$	Dependencies within detection based on pair type

Note. SD = standard deviation; MVN = multivariate normal distribution; $\mathcal{N}(\mu, \sigma^2)$ = normal distribution with mean μ and variance σ^2; $\mathcal{U}(a, b)$ = uniform distribution with support $x \in [a, b]$; Φ = probit transformation. LKJ is a distribution described in Lewandowski et al. (2009). Within the transformations, X is used to represent the respective untransformed parameter.

[a]Depends on corresponding group-level parameter.

Table S 2

Mean parameter values (M), standard deviations (SD), and the 95% posterior interval (95% PI) for the best-fitting general recognition theory (GRT) and the two-high threshold model (2HTM) for Experiments 1 and 2

		Experiment 1		Experiment 2	
		M (SD)	95% PI	M (SD)	95% PI
GRT	μ_{old_s}	2.20 (0.27)	[1.75, 2.80]		
	c_s	0.94 (0.05)	[0.85, 1.04]		
	μ_{old_p}	1.78 (0.20)	[1.44, 2.24]	1.90 (0.20)	[1.54, 2.34]
	μ_{spill}	0.08 (0.03)	[0.02, 0.15]	0.29 (0.07)	[0.16, 0.42]
	ϱ	0.05 (0.03)	[-0.01, 0.11]	0.33 (0.07)	[0.19, 0.46]
	σ_{old}	1.79 (0.34)	[1.22, 2.57]	2.09 (0.30)	[1.55, 2.72]
	c_l	0.94 (0.05)	[0.85, 1.04]	0.98 (0.06)	[0.87, 1.10]
	c_r	1.01 (0.05)	[0.91, 1.12]	0.98 (0.06)	[0.87, 1.09]
	b_l			0.11 (0.03)	[0.04, 0.18]
	b_r			0.14 (0.04)	[0.07, 0.21]
2HTM	d_{o_s}	0.61 (0.03)	[0.55, 0.68]		
	g_s	0.39 (0.03)	[0.35, 0.46]		
	d_{o_p}	0.52 (0.05)	[0.44, 0.59]	0.45 (0.05)	[0.34, 0.54]
	d_n	0.50 (0.06)	[0.38, 0.61]	0.60 (0.05)	[0.51, 0.69]
	g_{lru}	0.39 (0.03)	[0.35, 0.46]	0.46 (0.03)	[0.40, 0.52]
	g_{lgn}	0.38 (0.03)	[0.32, 0.45]	0.43 (0.04)	[0.35, 0.51]
	g_{lgo}	0.38 (0.03)	[0.32, 0.45]	0.45 (0.04)	[0.37, 0.53]
	g_{rdn}	0.35 (0.04)	[0.26, 0.43]	0.38 (0.04)	[0.29, 0.46]
	g_{rdo}	0.45 (0.05)	[0.36, 0.56]	0.51 (0.05)	[0.41, 0.62]
	g_{ldn}	0.30 (0.05)	[0.20, 0.40]	0.41 (0.04)	[0.33, 0.50]
	g_{ldo}	0.41 (0.05)	[0.32, 0.51]	0.41 (0.04)	[0.33, 0.50]

Note. Empty cells denote parameters that have been fixed in the respective model. The parameter ρ was not included in the best-fitting two-high threshold models and is therefore not reported here. Cells in which the same parameter estimates are reported denote parameters that have been estimated with an equality constraint.

References

Ashby, F. G., & Townsend, J. T. (1986). Varieties of perceptual independence. *Psychological Review*, *93*(2), 154–179. https://doi.org/10.1037/0033-295X.93.2.154

Lewandowski, D., Kurowicka, D., & Joe, H. (2009). Generating random correlation matrices based on vines and extended onion method. *Journal of Multivariate Analysis*, *100*(9), 1989–2001. https://doi.org/10.1016/j.jmva.2009.04.008

Swets, J. A., Tanner, W. P. J., & Birdsall, T. G. (1961). Decision processes in perception. *Psychological Review*, *68*(5), 301–340. https://doi.org/10.1037/h0040547

Wagenmakers, E.-J., Lodewyckx, T., Kuriyal, H., & Grasman, R. (2010). Bayesian hypothesis testing for psychologists: A tutorial on the savage–dickey method. *Cognitive Psychology*, *60*(3), 158–189. https://doi.org/10.1016/j.cogpsych.2009.12.001
